# Cardiac conduction system and the electrocardiogram of the common hippopotamus (*Hippopotamus amphibius*)

**DOI:** 10.1113/EP092519

**Published:** 2025-03-28

**Authors:** Morten B. Thomsen, Peter Agger, Henrik Lauridsen, Vibeke Sødring Elbrønd, Camilla Rensch Davidsen, Emma Smedsgaard Byskov, Frederik Stig Scharling, Tobias Wang, Sara Andreia Rodrigues Abreu, Stamatios Alan Tahas, Carsten Grøndahl, Mads Frost Bertelsen, Kirstine Calloe

**Affiliations:** ^1^ Department of Biomedical Sciences University of Copenhagen Copenhagen Denmark; ^2^ Department of Clinical Medicine Aarhus University Aarhus Denmark; ^3^ Department of Veterinary and Animal Sciences University of Copenhagen Frederiksberg Denmark; ^4^ Section for Zoophysiology, Department of Biology Aarhus University Aarhus Denmark; ^5^ Copenhagen Zoo Frederiksberg Denmark

**Keywords:** cardiovascular system, conduction system, electrocardiogram, hippopotamus, Purkinje fibre

## Abstract

The common hippopotamus (*Hippopotamus amphibius*) shares a common terrestrial ancestor with whales (Cetacea) and has independently evolved similar physiological adaptations to their aquatic lifestyle. Although several studies have explored the electrical signalling in whale hearts, the understanding of the conduction system and electrical activation of the hippopotamus heart remains sparse. We set out to map the conduction system within the hippopotamus heart and determine the sequence of electrical activation, including the mean electrical axis of ventricular activation. ECGs were recorded from three anaesthetized hippopotamuses. Histological samples were collected from two of these animals and from an additional animal. The hearts of the hippopotamuses constituted ∼0.3% of body mass and as in whales, the hearts were situated more cranially in the thoracic cavity compared to most terrestial mammals, and were spanning from the first to the fourth intercostal space. The network of Purkinje fibre strands extended deep into the ventricular walls and consisted of large, ovoid cells. Orthogonal ECG recordings revealed a mean electrical axis pointing towards the neck of the animal, indicating that electrical activation takes place in an apex‐to‐base direction.

## Abstract (highlights)

- **What is the central question of this study?**
  In this study, we hypothesized that the mean electrical activation of the hippopotamus ventricles takes place in an apex‐to‐base direction.
- **What is the main finding and its importance?**
  We found that the Purkinje fibre network extends deep into the ventricular and septal wall, resulting in a mean electrical activation of the hippopotamus heart in the apex‐to‐base direction. This finding aligns with other mammals within the superorder Artiodactyla, where the mean electrical activation reflects the distribution of the Purkinje fibre network, rather than the ventricular mass as observed in Primates and Carnivora.

## 1 INTRODUCTION

The common hippopotamus (*Hippopotamus amphibius* Linnaeus, 1758; etymology: Latinized version of the Greek words *híppos* = horse and *potamós* = river, i.e., ‘horse of the river’) is an even‐toed ungulate (order Artiodactyla) encountered south of the Sahara Desert. The family Hippopotamidae share a common terrestrial ancestor with cetaceans (whales and dolphins). The two groups split ∼53 million years ago and evolved independently into the aquatic and semi‐aquatic species that live today (McGowen et al., 2020, 2014). The Cetacean ancestors became fully aquatic during the Eocene, >40 million years ago, whereas the adaptations to a semi‐aquatic lifestyle in the lineage of extant hippopotamuses probably occurred in the late Miocene, ∼10 million years ago (Boisserie et al., 2011). Hippopotamuses spend most of their day in or near bodies of water, straying away only at dusk, to graze on grasslands (Eltringham, 1999). There are several adaptations of hippopotamuses to this semi‐aquatic lifestyle: the eyes, ears and nostrils are placed high on their head, allowing them to breathe and stay aware of their surroundings while being mostly submerged; and the skin consists of a thin epidermis and a thick dermis with adipose tissue and capillary loops, which might play an important role in thermoregulation (Meyer, 2012; Springer et al., 2021). Notably, these adaptations to life in water have occurred independently in cetaceans and hippopotamuses (Springer et al., 2021).

Although the cardiac anatomy and function in cetaceans have been studied in some detail (e.g., Bickett et al., 2019; Bisaillon et al., 1987; Goldbogen et al., 2019; James et al., 1995; Meijler et al., 1992; Ono et al., 2009; Slijper, 1962), very little is known about cardiac function in hippopotamuses. Diving bradycardia has been reported, where diving was associated with a reduction in heart rate from 90–100 to 30–40 beats/min while the animals were submerged (Elsner, 1966), but the electrical activation of the hippopotamus heart has, to the best of our knowledge, not been investigated previously.

The ECG reflects electrical activation and deactivation of the heart. The P‐wave reflects depolarization of the atria, the QRS complex ventricular depolarization, and the T‐wave reflects ventricular repolarization. Given that the QRS complex reflects ventricular depolarization, QRS complexes can be used to determine the mean electrical axis (MEA), which reflects the average direction and magnitude of the depolarization wave through the ventricles. Based on data from domestic animals, Hamlin and Smith (1965) suggested that mammals can be grouped into two groups based on ventricular activation pattern. In Primates and Carnivora, the ventricles activate in a base‐to‐apex direction; the MEA reflects the cardiac anatomy and points towards the left ventricle and this has the largest mass. Given that the MEA in these animals reflects the ventricular mass, axis deviations can indicate a change in ventricular mass, such as left or right ventricular hypertrophy (Zipes & Jalife, 2013). In the second group, Perissodactyla and Artiodactyla, in contrast, the ventricles are activated in an apex‐to‐base direction, and the MEA is in the direction of the neck of the animal. It has been suggested that this difference in ventricular activation pattern reflects differences in the distribution of the Purkinje fibre network (Elbrønd et al., 2023; Hamlin & Smith, 1965). In this study, we hypothesized that in the hippopotamus heart, the overall electrical activation of the ventricles takes place in an apex‐to‐base direction, similar to domestic artiodactyls, and that the Purkinje fibre network extends into the ventricular and septal wall. Our aims were as follows: (1) to investigate the topography of the heart of the common hippopotamus and the histology of the cardiac conduction system; and (2) to record the ECG and determine the MEA of the ventricular depolarization process in anaesthetized hippopotamuses.

## 2 MATERIALS AND METHODS

### 2.1 Ethical approval

The collection of data during anaesthesia or pre‐euthanasia was approved by the Danish Animal Experiments Inspectorate (license number 2012‐15‐2934‐00280). All animals were captive born and from the Copenhagen Zoo, an institution accredited by the European Association of Zoos and Aquaria (EAZA). The hippopotamus enclosure consisted of an indoor and outdoor area, each with a land (indoors, 160 m^2^; outdoors, 540 m^2^) and water part (indoors, 220 m^2^; outdoors, 130 m^2^; combined total volume of 450 m^3^), with an extensive water filtration system. The indoor water temperature was maintained ≥15°C, and access to the outdoor enclosure was provided between March and November each year. The diet consisted of hay and freshly cut grass, supplemented with a commercial solution of fat‐soluble vitamins, vitamins C and E, and selenium. Fruit and vegetables were provided intermittently for husbandry or training purposes. A commercial salt lick was available at all times but seldom used by the animals.

Body masses were estimated by experienced animal care staff and, in the event of euthanasia, confirmed on post‐mortem examination. Study animals were as follows: Animal #1, a 20‐year‐old male (∼1800 kg); Animal #2, a 4‐year‐old male (550 kg); Animal #3, a 5‐year‐old female (726 kg); and Animal #4, a 4‐month‐old male (120 kg). Animal #1 was anaesthetized for dental treatment and electroejaculation. Animals #2 and #3 were euthanized on the grounds of population management (Bertelsen, 2019). Animal #4 was euthanized on the grounds of persistent lameness. ECGs were recorded from Animals #1–#3. Tissue samples were obtained from Animals #2–#4. In no case were animals anaesthetized or euthanized for the purpose of this study, and sampling was opportunistic.

The anaesthetic protocol was administered intramuscularly via dart in all animals and consisted of a multimodal combination that was adapted depending on the individual and the reason for the anaesthesia. Briefly, the induction protocol consisted of xylazine (140–200 µg/kg), detomidine (2–20 µg/kg or omitted), medetomidine (8–10 µg/kg or omitted), romifidine (70 µg/kg or omitted), butorphanol (30–70 µg/kg), methadone (30–70 µg/kg), acepromazine (15–45 µg/kg), midazolam (35 µg/kg or omitted) and ketamine (140 µg/kg or omitted). During anaesthesia, the animals breathed room air supplemented with O_2_ via a nasal cannula or endotracheal tube. The anaesthesia was supplemented as necessary with reduced doses of the multimodal combination used for induction or with intravenous boluses of propofol and/or ketamine. Where appropriate, analgesia was provided by administration of meloxicam (0.55 mg/kg) intramuscularly.

Euthanasia of Animal #4 was undertaken by intravenous delivery of pentobarbitone overdose. In Animals #2 and #3, this was undertaken by physical destruction of the brain via gunshot by highly skilled personnel and under safe and legal firearms use as detailed by the American Veterinary Medical Association guidelines (Leary et al., 2020). Handling of the animals complied with *Experimental Physiology*’s policies regarding animal experiments.

### 2.2 Electrocardiogram recordings

All ECG recordings were obtained using custom‐made subcutaneous electrodes. The electrodes were placed in two different configurations: Einthoven and orthogonal configurations. The Einthoven configuration was as follows. Lead I: the positive electrode on the left forelimb caudal to the triceps muscle proximal to the olecranon and the negative electrode on the right forelimb caudal to the triceps muscle. Lead II: the positive electrode on the lefthind limb proximal to the stifle and the negative electrode as described for lead I. Leads III, aVL, aVR and aVF were calculated from leads I and II. In the orthogonal configuration, the three electrode pairs were placed as follows. Lead X: the positive electrode on the left forelimb caudal to the triceps muscle proximal to the olecranon and the negative electrode on the right forelimb caudal to the triceps muscle proximal to the olecranon. Lead Y: the positive electrode on the left hindlimb proximal to the stifle and the negative electrode on the breast over the pectoral muscles. Lead Z: the positive electrode dorsally and proximally to the spinous process of the seventh vertebra, the negative electrode ventrally over the seventh sternebra. In both configurations, a ground electrode was placed on the right hindlimb above the stifle. In the orthogonal system, the three leads were placed approximately perpendicular to each other, with their axes intersecting the heart. Ideally, the electrical resistance between the electrode and the heart should be identical, but our recordings were not adjusted for this difference.

### 2.3 Anatomical study

The topography of the heart of each hippopotamus was determined by removing several ribs on the left side of the animal. Within 30 min of euthanasia, hearts were removed and rinsed with saline. Cardiac tissue samples were obtained from the sinus node, left and right auricle and atrial free walls, the atrioventricular node, interventricular septum, left and right ventricular free wall, and the moderator band (trabecula septomarginalis). All samples were fixed in a 4% neutral buffered formaldehyde (VWR Chemicals) for 48–72 h before being processed using an automated Axlab Excelsior AS with a 16 h series of graded ethanol, xylene baths followed by paraffin embedding. The paraffin blocks were cut into sections 2–3 µm thick and stained with Resorcin–Fuchsin stain with Picric Acid (Merck). Collagen stained red, elastic fibres dark purple or black, muscle fibres grey/yellow and nuclei black. For immunohistochemistry, sections were prepared by Protease XXIV treatment, rinsed in Tris‐buffered saline (TBS) followed by 15 min in 3% H_2_O_2_ in TBS and 5 min in Ultravision Protein Block (Ultravision One Detection System, Epredia). The primary antibody, a rabbit polyclonal IgG targeted towards connexin 43 (ab11370 Abcam, Cambridge, UK) was diluted 1:500 in TBS with 1% bovine serum albumin, and the sections were incubated overnight at 4°C. After washing, the sections were incubated in Ultravision One HRP Polymer for 30 min followed by TBS wash and aminoethyl carbazole solution for 10 min (Ultravision One Detection System, Epredia). The sections were rinsed in distilled water and stained with Mayer's Haematoxylin (Merck) for 30 s followed by washing in distilled water and mounted using glycerol–gelatin. All procedures were performed at the Histology Core Facility at the Department of Veterinary and Animal Sciences, University of Copenhagen. Images were acquired using a Leica DMR microscope (Leica Microsystems) and the Leica Application Suite v.4.10, Las Core (Leica Microsystems). Adobe Photoshop 2024 was used to prepare the images for publication.

## 3 RESULTS

### 3.1 Topography of the heart

**FIGURE 1:**
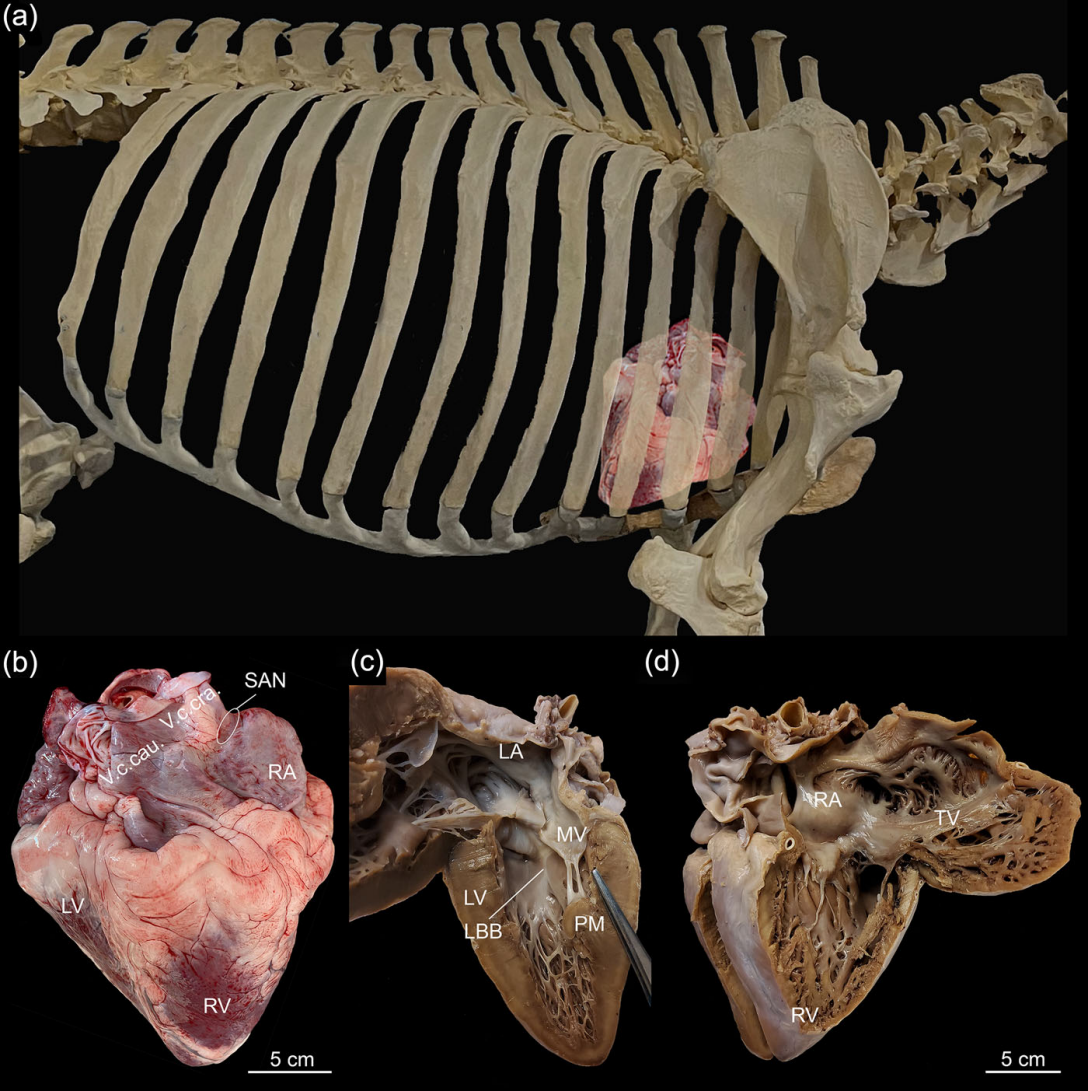
The hippopotamus heart. (a) The position of the heart in the thoracic cavity. The image is a modified version of an image of a *Hippopotamus amphibius* skeleton shared by SKELETONS: Museum of Osteology, Oklahoma City, OK, USA. https://commons.wikimedia.org/wiki/File:Hippopotamus_Skeleton_from_SKELETONS_Museum_of_Osteology.jpg. (b) A heart from an adult hippopotamus viewed from the right to demonstrate the position of the sinus node, right auricle, caudal caval vein, cranial caval vein, right ventricle and left ventricle. (c) The left septal wall of a fixed heart opened by incisions through the left atrium and through the left ventricle parallel to the septum. The left bundle branch is visible as a light subendocardial streak. (d) The right auricle and right ventricle opened, revealing the inside of the chambers of the same heart as in (b). The fixed heart (in c,d) was from a juvenile hippopotamus. Abbreviations: LA, left atrium; LBB, left bundle branch; LV, left ventricle; MV, mitral valve; PM, papillary muscle; RA, right auricle; LV, left ventricle; RV, right ventricle; SAN, sinus node; TV, tricuspid valve; V.c.cra., cranial caval vein; V.c.cau., caudal caval vein.

The heart and pericardium were situated in the mediastinum between the first and fourth intercostal space. The cranial long axis of the heart was almost resting on the sternum at an angle of ∼45° (Figure 1a). The apex pointed towards the caudal part of the sternum at the level of the costosternal junction of the fifth costa. All hearts had a single apex formed by the left ventricle (Figure 1b). The heart weight was determined for Animal #2 as 2.0 kg and for Animal #3 as 2.2 kg, corresponding to 0.3% of the total body mass. In the hearts of these animals, large amounts of adipose tissue were present at the coronary sulcus (Figure 1b). In Animal #2, a 4‐year‐old male, the aortic arch was bulbous, thin‐walled and distensible. The diameter was 45 mm, and the wall thickness was 3 mm. The aortic arch did not appear bulbous in the 4‐month‐old hippopotamus (Animal #4); the diameter of the aortic bulb was 30 mm and the wall thickness 6 mm. The appearance and diameter of the aortic bulb were not determined in Animal #3.

The atria and ventricles were opened, revealing a trabeculated endocardium and many free‐running Purkinje strands (Figure 1c,d).

### 3.2 Distribution and histology of the conduction system

**FIGURE 2:**
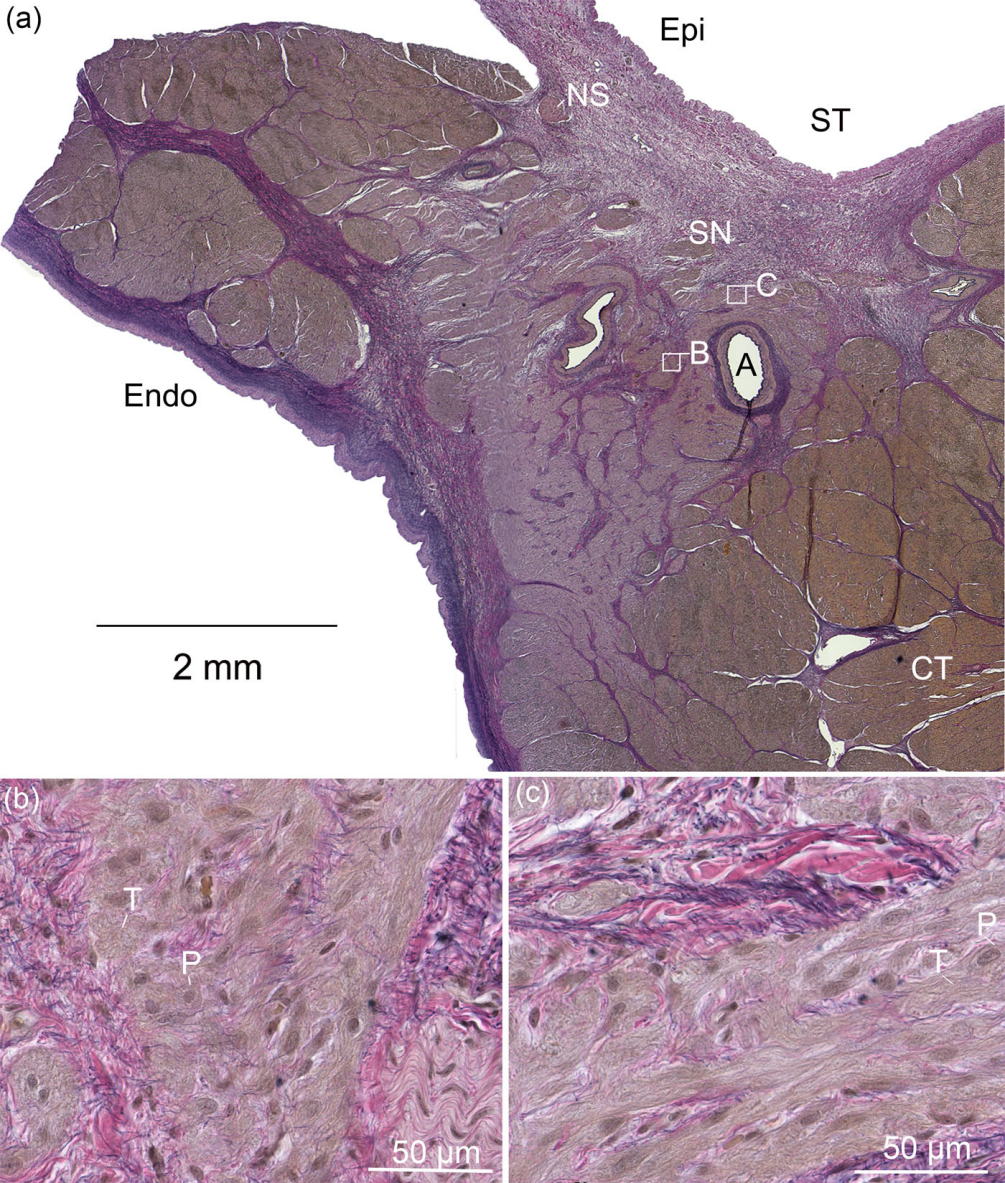
Sinus node from a hippopotamus. (a) Overview of the sinus node sinus node. The sinus node is found close to the epicardium, under the sulcus terminale. The sinus node contains arteries (one is indicated by A), nerve sections (NS), islets with P‐cells (P) and transitional cells (T). The white rectangles indicated by B and C are shown at higher magnification (in b and c, respectively). (b, c) P‐Cell clusters with short branching T‐cells. Resorcin Fuchsin stain. Abbreviations: CT, cristae terminale; Endo, endocardium; Epi, epicardium; SN, sinus node; ST, sulcus terminale.

The sinus node was located subepicardially and was faintly visible as a light streak close to the terminal crest in the right atrium. An overview of a transverse section is shown in Figure 2. The sinus node was infiltrated by an irregular network of connective tissue consisting of collagen and elastic fibres, and one or more central arteries were identified, with several adjacent nerves in their external tunica (tunica adventitia). Nodal cardiomyocytes were found in islets containing two major cell types: small round P‐cells with central distinct nuclei, pale cytoplasm and very few contractile elements. Short branching transitional cells (T‐cells) were interspersed between the P‐cells. The T‐cells were striated, suggesting the presence of contractile filaments. We did not observe Purkinje fibre‐like cells in the atria.

**FIGURE 3:**
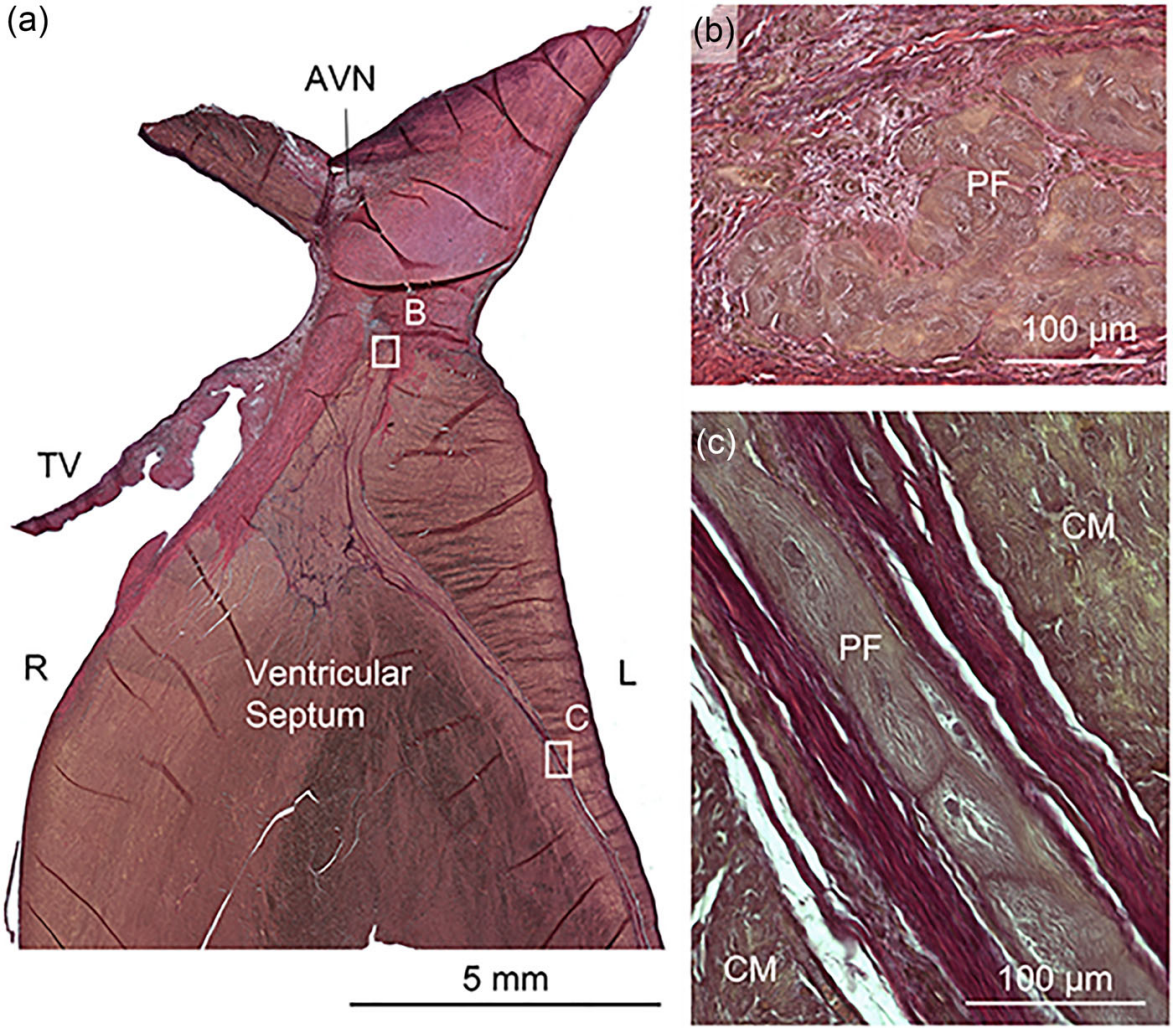
Bundle of His and left bundle branch. (a) Overview of the section containing parts of the atrial and ventricular septum, in addition to the septal tricuspid valve. (b) The penetrating bundle with relatively small round Purkinje fibres. (c) The left bundle branch with large Purkinje fibres arranged in parallel strands. Abbreviations: AVN, atrioventricular node; CM, cardiomyocytes; L, left; PF, Purkinje fibres; R, right; TV, tricuspid valve.

To isolate the atrioventricular node, the triangle of Koch was dissected. The triangle was outlined by the coronary sinus ostium, the septal leaflet of the tricuspid valve and the tendon of Todaro. The apex of the triangle was at the membranous septum. The atrioventricular node did not have distinct macroscopic characteristics and was found close to the orifice of the coronary sinus in the right atrium (Figure 3). The cells of the atrioventricular node were reminiscent of the atrial nodal cells, and ganglia with neuronal bodies were found close to the atrioventricular node.

The atria were separated from the ventricles by the fibrous body, and a penetrating conduction bundle from the atrioventricular node to the ventricular septum connected the atria and the ventricles electrically. The penetrating bundle consisted of smaller ovoid Purkinje cells with grey cytoplasm and a single, large spherical nucleus centrally. The number of contractile filaments was sparse in comparison to the surrounding working cardiomyocytes (Figure 3b). The bundle branches were visible macroscopically as pale streaks radiating over the left and right sides of the interventricular septum. The Purkinje cells in the bundle branches were larger and organized end‐to‐end in parallel strands. The contractile filaments were arranged longitudinally. In some cells, a central clearing was present, suggesting a washout of glycogen. The bundles were surrounded by thick collagenous sheaths (Figure 3c).

**FIGURE 4:**
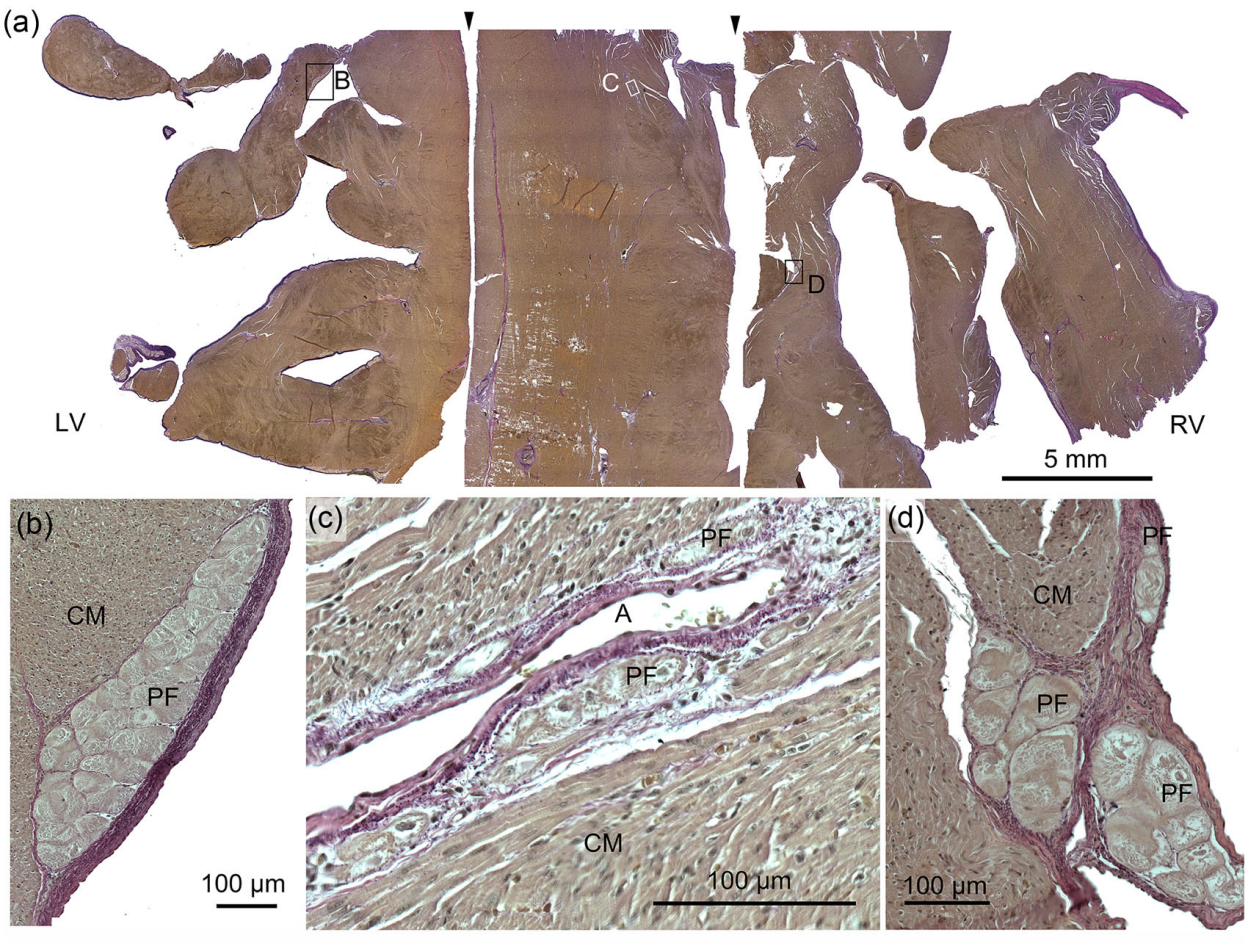
Transverse section of interventricular septum. (a) Overview of the septum consisting of three continuous samples; the left, middl, and right parts. The black arrowheads indicate the cut surfaces. (b–d) Enlargement of the framed areas (B, C and D) in (a) demonstrating subendocardial Purkinje fibres and PF near an artery (A) in the septal wall. Abbreviations: CM, cardiomyocytes; LV, left ventricle; PF, Purkinje fibres; RV, right ventricle.

The bundle branches turned into a network of trabeculae and an extended network of free‐running Purkinje strands (Figure 1c,d). A moderator band (trabeculum septomarginalis) was not observed. The Purkinje strands were mainly found subendocardially in the septal trabeculae, but some strands extended deep into the septal myocardium. These strands were often found adjacent to the connective tissue associated with blood vessels (Figure 4). The individual Purkinje cells were large and ovoid, with very few contractile filaments (Figure 4b–d).

**FIGURE 5:**
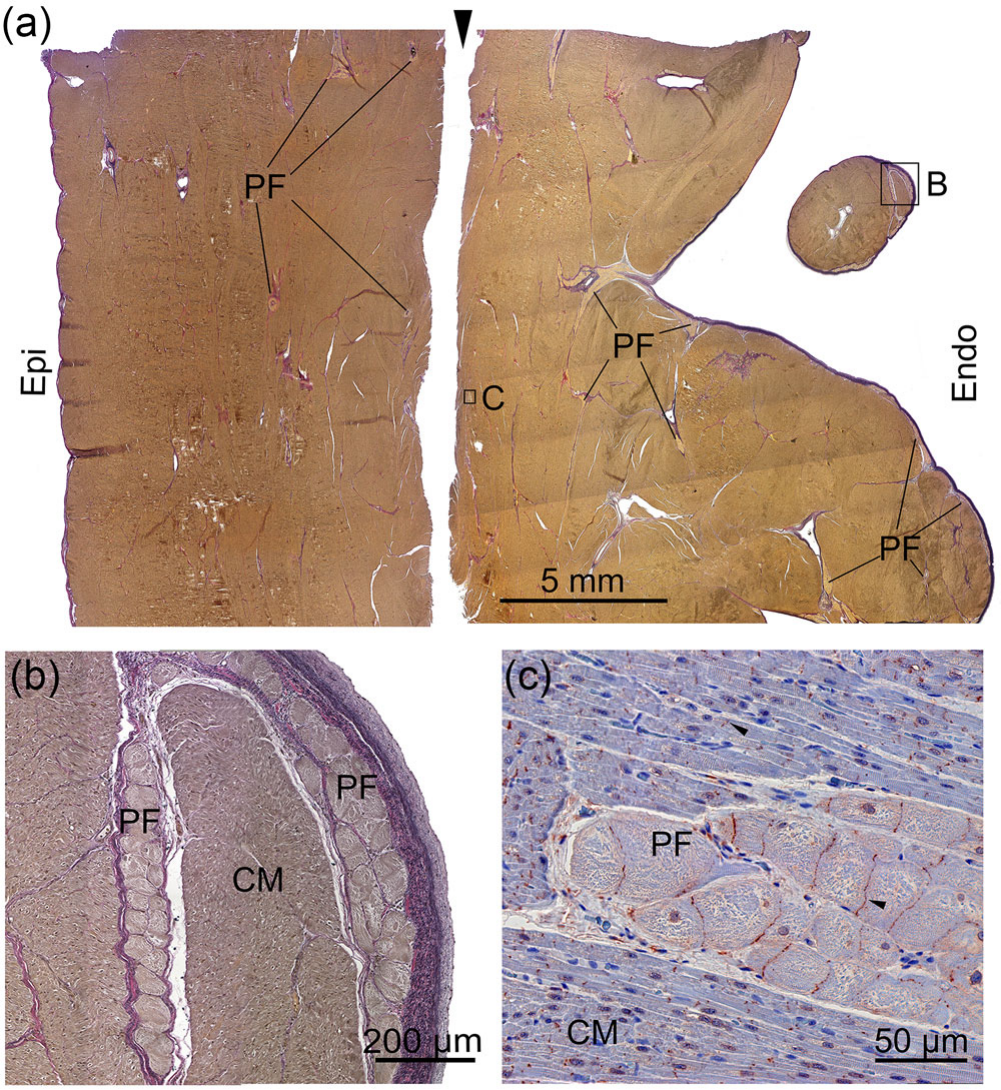
Transmural section of the left ventricular free wall from a hippopotamus. (a) An overview of the left ventricular wall. The two sections are consecutive. Purkinje fibre strands were found in the subendocardium, but also deep within the myocardium. The black arrowheads indicate the cut surface between the two sections. (b) Enlargement of the framed area (B) in (a) from a trabecula with Purkinje fibres located subendocardially and in the muscle tissue. (c) Enlargement of the framed area (C) in (a), showing that connexin 43 was expressed in the intercalated discs in the working myocardium and between the Purkinje fibres, as indicated by arrowheads. Immunohistochemistry with anti‐connexin 43 and Mayer's Haematoxylin. Abbreviations: CM, cardiomyocytes; Endo, endocardium; Epi, epicardium; PF, Purkinje fibres.

The Purkinje fibre network extended to the left and right ventricular free walls. As in the septum, the Purkinje fibre network was identified subendocardially and had extensions deep into the free walls (Figure 5). The majority of these intramural Purkinje strands in the mid‐myocardium were found in connection with collagenous tissue surrounding blood vessels. The morphology of the Purkinje cells in the free walls was similar to that of septal Purkinje cells. The intercalated discs of both Purkinje fibres and the working myocardium were positive for connexin 43 (Figure 5c).

### 3.3 Cardiac electrical activation

**FIGURE 6:**
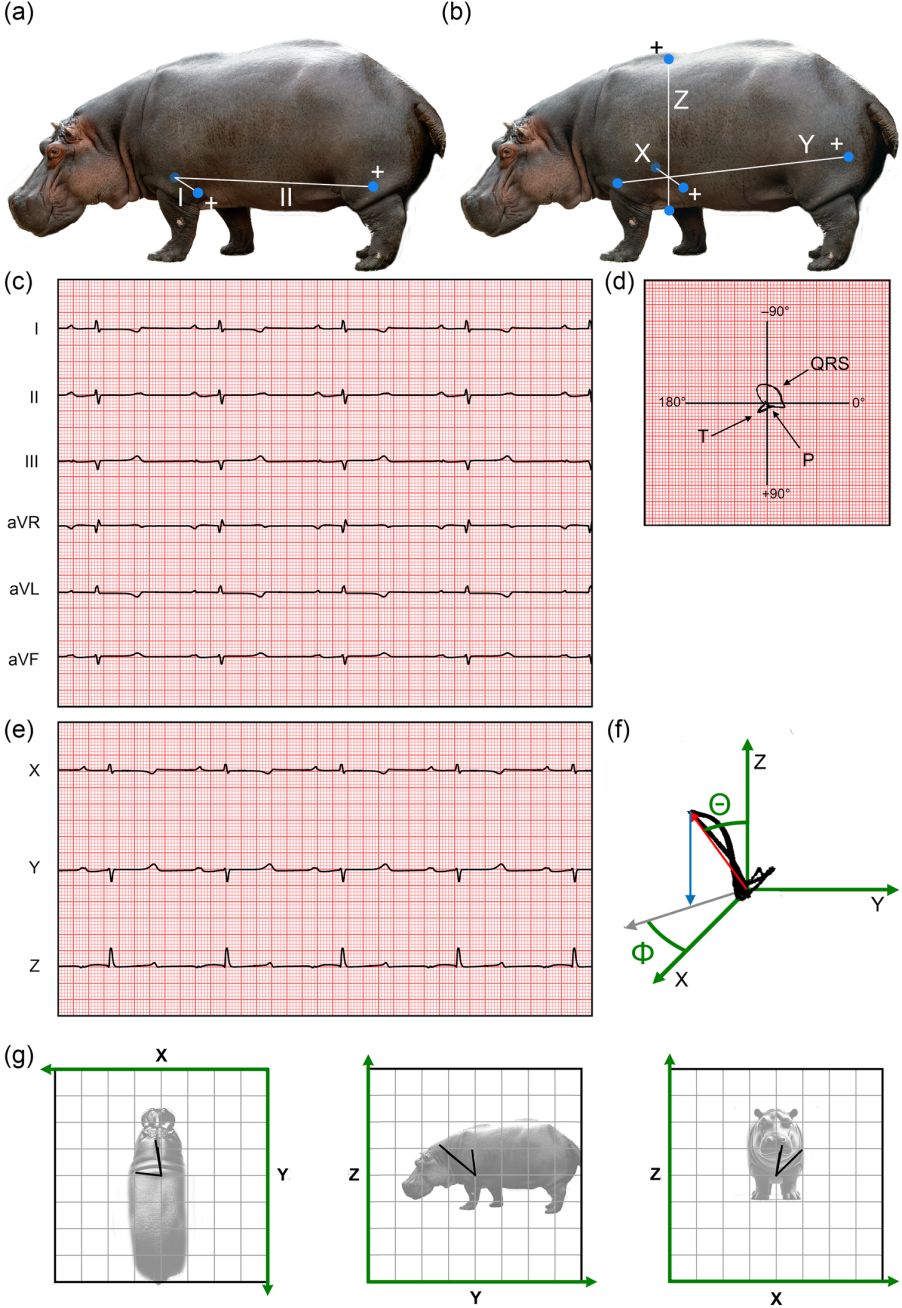
Electrical activation of the heart of the hippopotamus. (a) Position of electrodes in the Einthoven configuration. A ground electrode (not shown) was placed on the right hindlimb. (b) Position of electrodes in the orthogonal XYZ configuration. The positive electrode is indicated by (+). The recording speed was 25 mm/s and the amplitude 1 mV/cm in both configurations. (c) ECG in the Einthoven configuration. Note the low voltage of the QRS complexes. (d) Two‐dimensional vector loops of the entire 7 s recording shown in (c). Lead aVF is plotted against lead I. 0.5 mV/cm. (e) ECG obtained in the three‐dimensional orthogonal configuration. (f) Three‐dimensional vector loops based on ECG complexes recorded in the orthogonal configuration in (e). The mean electrical axis is indicated as a red arrow. The elevation, which is the angle between the mean electrical axis and the *z*‐axis running in a ventral–dorsal direction, is indicated by Θ, whereas the azimuth, which is the angle between the *x*‐axis and the projection of the mean electrical axis on the horizontal plane (grey arrow), is indicated by Φ. (g) Projections of the mean electrical axis on the XY, YZ and XZ planes, respectively, from three adult hippopotamuses. All axes are 4 mV long; all vectors originate at (0,0).

ECGs were obtained in Einthoven's configuration and in an orthogonal configuration (Figure 6a,b). The heart rate was 34 ± 9 beats/min (mean ± SD) (Table 1). In the Einthoven configuration, the amplitude of ECG deflections was small. In lead II, the P‐waves were positive deflections, the QRS complex exhibited an RS morphology, and the T‐waves were positive (Figure 6c). The durations of the different intervals are shown in Table 1. In the orthogonal configuration, the amplitude of the R‐wave was large and positive in the Z lead (0.47 ± 0.05 mV), suggesting a predominant electrical activation of the ventricles in an apex‐to‐base direction (Figure 6e). The amplitude of the maximal vector, calculated from the XYZ‐plot, was 0.57 ± 0.10 mV. The elevation (Θ; the angle between the vector and the *z*‐axis) was 27° ± 12°, and the azimuth (Φ; the angle between the *x*‐axis and the projection of the vector in the XY‐plane) was −57° ± 44° (*n* = 3; Figure 6f,g).

**TABLE 1:** ECG intervals from three anaesthetized hippopotamuses, Animals #1–#3.

Parameter	Value
Heart rate, beats/min	34 ± 9
RR interval, ms	1868 ± 574
PR interval, ms	414 ± 56
QRS interval, ms	104 ± 15
QT interval, ms	675 ± 37

Results are based on lead II and shown as the mean ± SD; *n* = 3.

## 4 DISCUSSION

The cranial position of the hippopotamus heart between the first and fourth intercostal spaces is in agreement with a previous report (Heinrichs & Wissdorf, 1988). This resembles the topography of the cetacean heart (Slijper, 1962), whereas the heart is typically found between the fourth and sixth intercostal spaces in terrestrial mammals (König & Liebich, 2020). Whales and hippopotamuses also share a barrel‐shaped thorax with lungs in a more dorsal and cranial position, which is likely to serve to balance buoyancy, hence their position when in the water (Slijper, 1962). Many cetaceans have a bifid ventricle, where the left and the right ventricles form their own independent apex (Rowlatt, 1981, 1990). The hippopotamus hearts in our study had the typical mammalian single apex formed by the left ventricle. A relative heart mass of 0.33% was found based on two subadult animals that had not grown to full size, but the relative heart mass is in agreement with a previous report based on two wild‐living hippopotamuses from Maji Moto Camp, Africa (Crile & Quiring, 1940). This relative heart mass is considerably smaller than in most mammalian species, in which the heart typically constitutes ∼0.5%–0.6% of the total body mass (Crile & Quiring, 1940; Seymour & Blaylock, 2000). Given that a similar heart‐to‐body‐mass relationship was found in the wild hippopotamuses (Crile & Quiring, 1940), our findings are unlikely to be associated with the study animals being captive bred, with a lower activity level and, presumably, better nutritional condition.

The aorta of the 4‐year‐old male had a thin‐walled and distensible bulbous aortic arch that resembled the highly compliant aortic arch found in whales and seals (Shadwick & Gosline, 1994, 1995). This provides for an arterial Windkessel effect, whereby compliance enables distension when the stroke volume is expelled during systole. Thus, during the prolonged diastole associated with diving bradycardia, peripheral blood flow is maintained by the elastic recoil of the bulbous aortic arch. The aortic arch did not appear bulbous in the 4‐month‐old hippopotamus and was not noted as bulbous in two previous reports describing the anatomy of hippopotamus hearts (Frick, 1956; Heinrichs & Wissdorf, 1988). The animal examined by Heinrichs and Wissdorf was 6 months old (Heinrichs & Wissdorf, 1988) and it is possible that the presence of a bulbous aortic arch is age dependent. As our finding is based on one animal, it could be a rare incidence or, potentially, a pathological condition.

The overall structure and distribution of the conduction system, including the positions and cytology of the sinus node and atrioventricular node, of the hippopotamus heart were similar to those of whales and other artiodactylsas (James, 2002; Ono et al., 2009; Pfeiffer, 1990). The round P‐cells of the sinus node and atrioventricular node were small and connected to small branching T‐cells. The P‐cells are thought to function as the pacemakers of the heart, whereas the T‐cells might play a role in synchronizing the pacing between P‐cells. In the septal and ventricular walls, the Purkinje fibres were large, ovoid, and easy to identify owing to their characteristic morphology with very few myofibrils. Very large Purkinje fibres of a similar morphology have been described in cetaceans (Ono et al., 2009; Pfeiffer, 1990). The Purkinje fibre network was mainly found subendocardially on the trabecular muscles of the septum and ventricles, but the network also extended deep into the septal and free ventricular walls. This is consistent with intramural Purkinje fibres previously reported in whales and terrestrial artiodactyls (Calloe, 2019; De Almeida et al., 2015; Elbrønd et al., 2023; Ono et al., 2009). The Purkinje fibres were arranged in strands ensheathed by collagen fibres, offering both mechanical protection and electrical shielding, and the intramural Purkinje strands were often found in connection to blood vessels. The intramural Purkinje network ensures a rapid and uniform electrical activation of the heart (Hamlin & Smith, 1965). In agreement with the Purkinje fibre network being optimized for rapid electrical transmission, we found expression of high‐conductance gap junctions formed by connexin 43, which were also expressed in the working myocardium.

### 4.1 Electrocardiogram

The average heart rate of the anaesthetized hippopotamuses (34 beats/min; see Table 1) was considerably lower than that of 90–100 beats/min in awake hippopotamuses but similar to the diving values of 30–40 beats/min measured by Elsner (1966). It has been reported that anaesthesia and, in particular, opioids might trigger a dive response with subsequent bradycardia and apnoea (West et al., 2007); however, several anaesthetics, including α_2_‐adrenergic receptor agonists, which were the mainstay of the anaesthetic protocols used in the animals of this study, can have negative inotropic and chronotropic effects, resulting in lower heart rate and blood pressure (Muir, 2015).

The PR interval, reflecting conduction time through the atrioventricular node, was 414 ± 56 ms. Although heart mass increases with body mass and heart rate decreases, the PR interval is remarkably constant at ∼350–400 ms for larger animals, including cetaceans, elephants and horses. The prolonged PR interval found in this study could be an effect of anaesthesia. The QRS and QT intervals scale with body mass (Meijler et al., 1992) and, interestingly, differences in the allometric relationships between body mass and ECG intervals for terrestrial and marine mammals have been reported (Storlund et al., 2021). In general, marine mammals have wider QRS complexes and relatively shorter QT intervals compared with terrestrial animals of similar sizes. At present, owing to the low number of individuals, we cannot conclude whether the allometric relationships between body mass and ECG intervals for hippopotamuses comply with those of terrestrial or marine mammals. Age, physical activity and anaesthesia are also likely to affect ECG intervals.

The amplitudes of the deflections in Einthoven's configuration were small. The largest R waves were found in lead Z of the orthogonal configuration, and the MEA was towards the neck of the animal, reflecting a ventricular activation in an apex‐to‐base direction. This is similar to the activation pattern reported for domestic ungulates (Artiodactyla and Perissodactyla) (Elbrønd et al., 2023; Hamlin & Smith, 1965). It has been proposed that the apex‐to‐base MEA is caused by an intramural component of the Purkinje fibre network (Elbrønd et al., 2023; Hamlin & Smith, 1965). An intramural Purkinje fibre network ensures simultaneous electrical activation across the ventricular layers and, thereby, a coordinated transmural contraction. In contrast, the MEA has a base‐to‐apex direction in Primates and Carnivora, in which the Purkinje fibre network is exclusively located subendocardially (James, 2002; Ono et al., 2009; Scharling et al., 2024). The depolarization wave spreads through the working cardiomyocytes to the mid‐ and epicardium, resulting in a delay of activation in these layers owing to the low conduction velocity of the myocardium (Boukens et al., 2017; Calloe et al., 2008). The activation delay has been suggested to be compensated by a transmural gradient in the early repolarization phase of the cardiac action potential. This phase determines the timing of calcium release and thereby the excitation–contraction coupling (Calloe, 2019; Cordeiro et al., 2004; Sah et al., 2003). This suggests that the Purkinje fibre distribution determines the pattern of electrical activation of the ventricles and the MEA in mammalian species.

In cetaceans, ECGs are typically recorded using a single device attached to the animal with suction cups, allowing the animal to swim and dive (Bickett et al., 2019; Goldbogen et al., 2019; Meijler et al., 1992; Williams et al., 2017). These devices have both positive and negative electrodes with minimal inter‐electrode distance. This configuration does not allow for the evaluation of a cardiac electrical axis. A modified six‐lead ECGs in an oblique plane with a negative lead placed on the dorsal midline and a positive lead placed in the left axillary space of dolphins (Harms et al., 2013; Yaw et al., 2018) provides ECGs with an rS configuration in the lead closest to the presumed base‐to‐apex axis. This agrees with a MEA in the apex‐to‐base direction.

## 5 CONCLUSION

In line with our hypotheses, the overall electrical activation of the hippopotamus heart occurs in the apex‐to‐base direction, and the Purkinje fibre network extends deep into the ventricular and septal walls. Similar to other mammals within the superorder Artiodactyla, the MEA is likely to reflect the distribution of the Purkinje fibre network, rather than reflecting the ventricular mass as in Primate*s* and Carnivora.

## AUTHOR CONTRIBUTIONS

ECG and tissue sampling: Morten B. Thomsen, Peter Agger, Henrik Lauridsen, Vibeke Sødring Elbrønd, Camilla Rensch Davidsen, Emma Smedsgaard Byskov, Frederik Stig Scharling, Sara Andreia Rodrigues Abreu, Stamatios Alan Tahas, Carsten Grøndahl, Mads Frost Bertelsen and Kirstine Calloe. Drafting the manuscript: Morten B. Thomsen and Kirstine Calloe. All authors have critically reviewed the manuscript and approved the final version. They all agree to be accountable for all aspects of the work in ensuring that questions related to the accuracy or integrity of any part of the work are appropriately investigated and resolved. All persons designated as authors qualify for authorship, and all those who qualify for authorship are listed.

## CONFLICT OF INTEREST

None declared.

## Data Availability

Data are available from the corresponding author upon request.
